# Detection of severity in Alzheimer's disease (AD) using computational modeling

**DOI:** 10.6026/97320630014259

**Published:** 2018-05-31

**Authors:** Hyunjo Kim

**Affiliations:** 1 Department of Life Science, University of Gachon, Seungnam, Kyeonggido, Korea; 2 Medical Informatics Department of Ajou Medical Center, South Korea

**Keywords:** Alzheimer's disease, mini mental state examination, acetyl cholin esterase inhibitors, MRI, K-means: algorithm simulation

## Abstract

The prevalent cause of dementia - Alzheimer's disease (AD) is characterized by an early cholinergic deficit that is in part responsible for the cognitive deficits (especially memory and attention defects). Prolonged AD leads to moderate-to-severe AD, which is one of the leading causes of death. Placebo-controlled, randomized clinical trials have shown significant effects of Acetyl cholin esterase inhibitors (ChEIs) on function, cognition, activities of daily living (ADL) and behavioral symptoms in patients. Studies have shown comparable effects for ChEIs in patients with moderate-to-severe or mild AD. Setting a fixed measurement (e.g. a Mini-Mental State Examination score, as a 'when to stop treatment limit) for the disease is not clinically rational. Detection of changed regional cerebral blood flow in mild cognitive impairment and early AD by perfusion-weighted magnetic resonance imaging has been a challenge. The utility of perfusion-weighted magnetic resonance imaging (PW-MRI) for detecting changes in regional cerebral blood flow (rCBF) in patients with mild cognitive impairment (MCI) and early AD was evaluated. We describe a computer aided prediction model to determine the severity of AD using known data in literature. We designed an automated system for the determination of AD severity. It is used to predict the clinical cases and conditions with disagreements from specialist. The model described is useful in clinical practice to validate diagnosis.

## Background

Alzheimer's disease (AD) and its related dementia have shown an alarming rise in the global population. Although considerable efforts have been made to develop effective therapeutic agents for AD therapy, drug development has not met significant clinical success [1]. Current pharmaco-therapy of AD is limited to cholinesterase inhibitors and the N-methyl- D-aspartate antagonist. Considerable research is underway to develop new agents for the management of AD. Since amyloid- β (Aβ) has been implicated in AD pathogenesis, the use of β secretase inhibitors as well as immunotherapy against Aβ has been studied [2].

Baseline measures, such as degree of cognitive impairment, rate of disease progression, older age, smoking habit, and the presence of concurrent vascular risk factors, are able to affect the clinical response. Some of these parameters (age, cerebrovascular disease, as well as hippocampal atrophy) may act through structural mechanisms, smoking through chemical molecules [3]. The presence of sub-cortical vascular lesions has been reported not to significantly affect the response to ChEIs [4]. Another question at issue is the reproducibility, in a "real world" setting, of the results achieved in controlled clinical trials, where the selection of AD patients, based on very restrictive criteria, makes the cohorts more homogeneous and generally younger with respect to everyday clinical practice [5]. These observations show the need for a novel approximation approach to posterior expectations of real valued functions, given observed data, which may allow clinical practitioners to obtain a clearer view of the expected net benefit for treatment. Therefore, encouraging clinical data collection from patients out of randomized clinical trials will give biostatisticians the information needed to build an algorithm [6, 7]. We focus on AD neuro-imaging initiative studies published between 2011 and March 2014 for which structural MRI was the outcome measure. It is of interest to document the relationships of structural MRI measures to cognition.

Several studies report correlations between regional brain volume or atrophy and various types of cognitive tests. As expected, memory measures correlate best with temporal lobe structures, whereas executive function and general cognitive functioning measures typically correlate more strongly with global measures, such as whole-brain atrophy, ventricular enlargement, and cortical thickness across multiple brain regions [8, 9, 10, 11].

The ability to precisely identify the stage of disease, predict the rate of disease progression, and accurately measure the outcomes of potential therapies is critical to the successful management of Alzheimer's disease (AD). The classical characterization of lateonset AD progression is a time-ordered succession of 3 stages: normal (N), mild cognitive Impairment (MCI), and AD. Physical measurements of disease progression, that is, MRIs, are used to classify patients into these 3 stages, but it has been challenging to reliably define finer stages of the disease [12, 13].

### Description

We removed the effects of normal aging from the MRI data during pre-processing. The rationale for this is related to the fact that the effects of normal aging on the brain are likely to be similar (equally directed) with the effects of AD, which can lead to an overlap between the brain atrophies caused by age and AD. This would bring a possible confounding effect on the estimation of disease-specific differences.

### Relevance of structural imaging

Patients with significant cognitive impairment but who do not meet criteria for dementia are at increased risk for developing AD, and a number of approaches can be considered in order to achieve an early diagnosis. Although screening neuropsychological tests are necessary to recognize and monitor these at-risk subjects, there is no perfectly accurate cognitive marker of early AD identified to date [14]. Moreover, cognitive performances depend not only on age and education but also on mood and attention at the time of testing and thus lack wide general features. Likewise, the concentration of tau protein and amyloid β (Aβ) in the cerebral-spinal fluid (CSF) appears to have some diagnostic value in probable AD, but it is an invasive procedure and its value for predicting AD has received only little attention thus far [15].

Perfusion magnetic resonance imaging (MRI) can be used to assess cerebral hemodynamic parameters for non-invasive diagnosis and staging of disease and for treatment monitoring. This method involves monitoring of rapid changes in signal intensity over time for a tracer passing though the capillary bed. Quantitative analysis using dynamic susceptibility contrast (DSC) MRI perfusion requires determination of the arterial input function (AIF), which is the concentration of the contrast agent over time in a brain-feeding artery [16, 17]. It is used in the de-convolution of tissue time-concentration curves to obtain hemodynamic maps of cerebral blood flow (CBF), cerebral blood volume (CBV), and mean transit time (MTT) [18, 19, 20, 21, 22, 23, 24, 25]. Thus, AIF profile has a profound effect on final calculation of cerebral blood parameters.

## Methodology

### Relationships of structural MRI to cognition

A MRI ultimately must be linked to cognition or must predict future changes in cognition. Many studies have sought to establish a link between various AD biomarkers and cognition at different disease stages. We focus on studies that related structural MRI measures to cognitive change.

### Basics of K-Means and Fuzzy C-Means

This section briefly explains about the algorithms related to kmeans and fuzzy c-means clustering techniques [26, 27].

### Algorithmic for K-Means Clustering

K-means clustering aims to partition n observations into k clusters in which each observation belongs to the cluster with the nearest mean; Let X = {x1, x2, x3, ..., xn} be the set of data points and V ={v1, v2, ..., vc} be the set of centers.

### Algorithm for Fuzzy C Means

The fuzzy c-means algorithm is as same as the k-means algorithm. The algorithm minimizes intra-cluster variance, but has the same problems as k- means, the minimum is a local minimum, and the results are based on the initial choice of weights [28]. The expectation-maximization algorithm is a more statistically formalized method, which includes some of these ideas: partial membership in classes. It has better convergence properties and is in general preferred to fuzzy-c-means.

Medical imaging is the special method and process used to create images of the human body for clinical purposes (medical procedures seeking to reveal, diagnose, or examine disease) or medical science (including the study of normal anatomy and physiology).

Measurement and recording techniques which are not primarily designed to produce images, such as electroencephalography (EEG), etc., but which produce data susceptible to be represented as maps (i.e., containing positional information), can be seen as forms of medical imaging [29].

### Image Segmentation Approach

**Figure 2:**
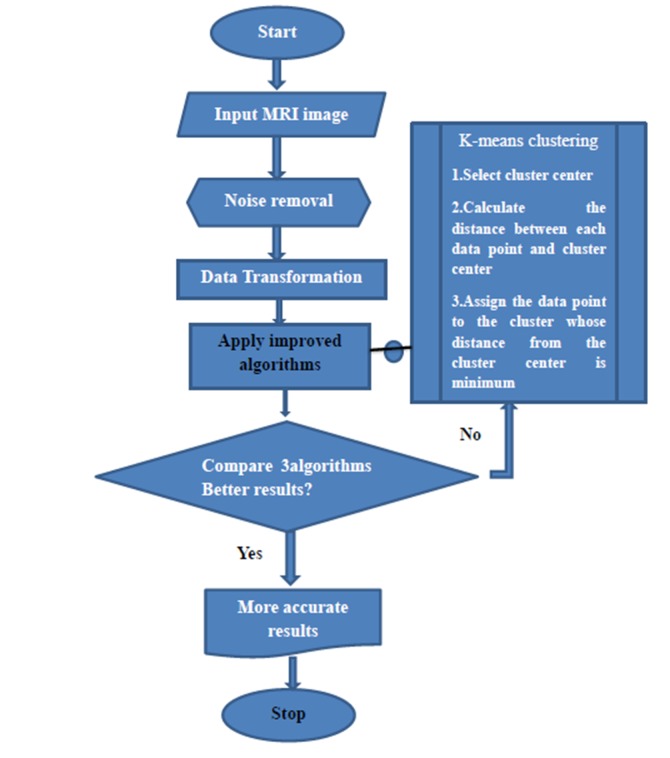
Flow chart to represent the data flow.

Image segmentation approach follows steps as reading the image, removing the noise from the image, data transformation, data normalization and comparative analysis of algorithms (Figure 2).

### Data Manipulation and analysis: Simulation Data

Perfusion magnetic resonance imaging (MRI) can be used to assess cerebral hemodynamic parameters for non-invasive diagnosis and staging of disease and for treatment monitoring. This method involves monitoring of rapid changes in signal intensity over time for a tracer passing though the capillary bed. Quantitative analysis using dynamic susceptibility contrast (DSC) MRI perfusion requires determination of the arterial input function (AIF), which is the concentration of the contrast agent over time in a brain-feeding artery [16, 17].

It is used in the de-convolution of tissue time-concentration curves to obtain hemodynamic maps of cerebral blood flow (CBF), cerebral blood volume (CBV), and mean transit time (MTT). Thus, AIF profile has a profound effect on final calculation of cerebral blood. According to the predetermined steps, AIFs would be obtained for each participant using the Kmeans in clinical data.

All the experiments were carried out on an off-line personal computer (Intel(R) Core(TM) i3 M350 CPU processor, 2.27 GHz operating frequency, 4.0 GB RAM memory capacity, Microsoft window 7 home premiums, 64-bit operating system). Algorithm was developed for comparison between FCM and K-means clustering using both simulated data and clinical data. The simulation was set up as reported by an automatic selection of arterial input function on dynamic contrast-enhanced MR images of computer methods programs (see supplementary data).

### Predicting Cognitive Decline

AD is a degenerative brain disease and the most common cause of dementia. It is characterized by a gradual and relentless progression of cognitive, functional and behavioral deficits [30]. The prevalence of Alzheimer's disease increases exponentially with age from around 1.5% at age of 65 years then doubling every 4 years to around 30% at the age of 80 years [31]. The exact pathophysiology of AD is not known. However, a neuronal cholinergic deficit can be demonstrated in the early phases of the disease [32]. The cholinergic system is a ubiquitous activating neurotransmitter system in the brain involved in higher cognitive functions such as memory and attention [32]. Mild cognitive impairment is a transitional stage between age-related cognitive decline and AD. For the effective treatment of AD, it is important to identify MCI patients at high risk for conversion to AD. In this study, we presented a magnetic resonance imaging (MRI)-based method for predicting the MCI-to-AD conversion prior to one to three years before the clinical diagnosis.

We developed a MRI based biomarker model of MCI-to-AD conversion using semi-supervised learning and then integrated it with age and cognitive measures about the subjects using a supervised learning algorithm resulting in aggregate biomarkers. The characteristics of the method used for learning the biomarkers are as follows: 1) a semi- supervised learning method (low density separation) for the construction of MRI biomarker as opposed to more typical supervised methods; 2) a feature selection on MRI data from AD subjects and normal controls without using data from MCI subjects via regularized logistic regression; 3) removal of the aging effects from the MRI data before the classifier training to prevent possible confounding between AD and age related atrophies; and 4) construction of the aggregate biomarker by first learning a separate MRI biomarker and then combining it with age and cognitive measures about the MCI subjects at the baseline by applying a random forest classifier. We experimentally demonstrated the added value of these characteristics in predicting the MCI-to-AD conversion on data obtained from the Alzheimer's disease Neuroimaging Initiative (ADNI) database. With the ADNI data, the MRI biomarker achieved a 10-fold cross-validated area under the receiver operating characteristic curve (AUC) of 0.7661 in discriminating progressive MCI patients (pMCI) from stable MCI patients (sMCI). The aggregate biomarker based on MRI data together with baseline cognitive measurements and age achieved a 10-fold cross-validated AUC score of 0.9020 in discriminating pMCI from sMCI. The results presented in this study demonstrate the potential of the suggested approach for early AD diagnosis and an important role of MRI in the MCI-to- AD conversion prediction. However, it is evident based on our results that combining MRI data with cognitive test results improved the accuracy of the MCI-to-AD conversion prediction.

Thus, K-means-based AIF determination might be less affected by mixing of the arterial signal with signals from surrounding tissue [32, 36], so the resulting AIF approaches optimality. AUC was higher for the K-means method than for FCM and was closer to the true AIF for simulation data. However, the K-means-FCM difference was significant for clinical data. This indicates that AIF determination based on the K-means method is affected by minimal partial volume averaging [32]. The higher peak and larger integrated bolus curve for the K-means-based AIF indicate that this method yields the measurements more close to true AIFs [32], so it should facilitate more accurate quantitative determination of CBF, CBV, and MTT. Each algorithm was executed 50 times for the same batch of data for comparison. The results reveal better reproducibility for K-means clustering than for FCM analysis. It is known that erratic AIFs lead to nonreproducible quantification of cerebral parameters, which undermines the diagnosis and tracking of the disease. Thus, compared to FCM clustering, the K-means method is preferable for AIF determination. The results demonstrated that the mean execution time was relatively longer compared with the K-means method and the difference was significant. In current PACS environments, the total execution time required for radiodiagnosis includes the duration of image downloading from the PACS server, image post-processing on a local workstation, and image unloading to the PACS server. The entire operation process takes a few minutes to complete within ten minutes. Relative to the total duration of image manipulation in PACS settings, the extra time required for executing the K-means method compared with the FCM method is negligible. Thus, the extra execution time did not limit the use of the K-means method for AIF determination in clinical practice. It must be noted that there were three limitations in this method. The number of subjects participated in perfusion imaging is only 42 subjects for the statistical analysis. This limited number of cases might result in statistical uncertainty [37]. Therefore, it is necessary to increase the number of subjects in similar studies in the future. All the participants involved in this study were healthy and subjects with abnormalities were not included. Thus, the clinical efficacy was not validated for patients with neurological diseases, which means that it is necessary to further assess the feasibility and efficiency of this method by adding DSC images of abnormal cases with acute stroke, artery stenosis, and other abnormalities. We evaluated the two most widely used clustering algorithms, so it is still unclear whether there are significant differences among other clustering algorithms used for AIF detection. Thus, it is necessary to compare other types of clustering algorithms to identify the most suitable clustering method for AIF determination. In conclusion, the K-means method yields more accurate and reproducible AIF results compared to FCM cluster analysis. The execution time is longer for the K-means method than for FCM with robust and accurate follow-up hemodynamic maps.

## Conclusion

We describe an automated algorithm combined with a learning method using MRI image features to predict the severity of AD.

## Conflict of Interest

The author declares no conflict of interest

## Supplementary data

Supplementary data

## Figures and Tables

**Figure 1:**
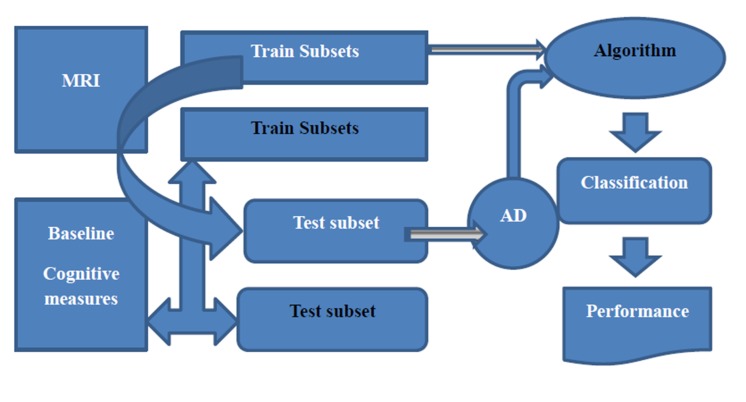
For computing the output of AD classifier for test subjects, the test subset is used in the algorithm and learning procedure without any label information (shown with whitegrayed arrow).
