# Supplementary material for: Detection of severity in Alzheimer's disease (AD) using computational modeling
Source: Bioinformation. 2018 May 31;14(5):259–64. doi: 10.6026/97320630014259 (PMC6077821; doi:10.6026/97320630014259)
Supplement: Supplementary data [file 97320630014259S1.pdf]

Supplementary Data:

The true AIF comprises the main peak CP(t) and a subsequent recirculation CR(t):

$$AIF = Cp(t) + Cr(t) \quad \text{— (1)}$$

$$Cp(t) = \begin{cases} 0_t & < t_0 \\ (t - t_0)^{\alpha} \exp\left(\frac{-t - t_0}{\beta}\right) & t = t_0 \end{cases} \quad \text{— (2)}$$

$$Cr(t) = \kappa\, Cp(t - t_d) \otimes \exp\left(\frac{-t}{tr}\right) \quad \text{— (3)}$$

Where t0 is the arrival time of contrast agent, α is a measure of in flow velocity steepness, b is the washout velocity, the symbol ''β'' represents the convolution operation, td is the delay between the principal peak and recirculation, tR is the time constant for the function accounting for recirculation dispersion, and k is a constant that ensures that the recirculation peak is the third part of the main peak, which closely approximates the contrast agent arrival time for our clinical perfusion data. The residue function R(t) was modeled using a gamma variation function to simulate the presence of bolus dispersion.

$$R(t) = t * \exp\left(\frac{-1}{\sqrt{MTT}}\right) \quad \text{—— (4)}$$

Where MTT equals the ratio of CBV to CBF. Then the relationship between contrast concentration C(t) and signal intensity S(t) was established using the following equations:

$$C(t) = \frac{\rho}{\kappa_H} CBF(AIF \otimes R(t)) \quad \text{—— (5)}$$

$$S(t) = S_0 * \exp(-\kappa_{VOX} * TE * C(t)) \quad \text{—— (6)}$$

During the scanning of perfusion images, some fluctuating curves were obtained because of shifts in voxels, PVEs, physiological pulsations, and other effects. These irregular curves would produce poor estimates of the true AIF. Thus, the following standard roughness measurement method was used and the rough percentage of the remaining curves with the largest integral values was excluded.

$$\Lambda(C) = \int_0^t (C^n(t))dt \quad \text{—— (7)}$$
